# Factors Likely to Affect Community Acceptance of a Malaria Vaccine in Two Districts of Ghana: A Qualitative Study

**DOI:** 10.1371/journal.pone.0109707

**Published:** 2014-10-15

**Authors:** Arantza Meñaca, Harry Tagbor, Rose Adjei, Constance Bart-Plange, Yvette Collymore, Antoinette Ba-Nguz, Kelsey Mertes, Allison Bingham

**Affiliations:** 1 Departmento de Antropología Social, Universidad Complutense de Madrid, Madrid, Spain; 2 Malaria in Pregnancy Group, Department of Community Health, School of Medical Sciences, Kwame Nkrumah University of Science and Technology, Kumasi, Ghana; 3 National Malaria Control Programme, Ghana Health Service, Accra, Ghana; 4 PATH Malaria Vaccine Initiative, Washington DC, United States of America; 5 PATH Malaria Vaccine Initiative, Nairobi, Kenya; 6 PATH Kenya, Kisumu, Kenya; The George Washington University Medical Center, United States of America

## Abstract

Malaria is a leading cause of morbidity and mortality among children in Ghana. As part of the effort to inform local and national decision-making in preparation for possible malaria vaccine introduction, this qualitative study explored community-level factors that could affect vaccine acceptance in Ghana and provides recommendations for a health communications strategy. The study was conducted in two purposively selected districts: the Ashanti and Upper East Regions. A total of 25 focus group discussions, 107 in-depth interviews, and 21 semi-structured observations at Child Welfare Clinics were conducted. Malaria was acknowledged to be one of the most common health problems among children. While mosquitoes were linked to the cause and bed nets were considered to be the main preventive method, participants acknowledged that no single measure prevented malaria. The communities highly valued vaccines and cited vaccination as the main motivation for taking children to Child Welfare Clinics. Nevertheless, knowledge of specific vaccines and what they do was limited. While communities accepted the idea of minor vaccine side effects, other side effects perceived to be more serious could deter families from taking children for vaccination, especially during vaccination campaigns. Attendance at Child Welfare Clinics after age nine months was limited. Observations at clinics revealed that while two different opportunities for counseling were offered, little attention was given to addressing mothers’ specific concerns and to answering questions related to child immunization. Positive community attitudes toward vaccines and the understanding that malaria prevention requires a comprehensive approach would support the introduction of a malaria vaccine. These attitudes are bolstered by a well-established child welfare program and the availability in Ghana of active, flexible structures for conveying health information to communities. At the same time, it would be important to improve the quality of Child Welfare Clinic services, particularly in relation to communication around vaccination.

## Introduction

Malaria is one of the main causes of mortality among children younger than five years in Africa, where approximately half a million children died from malaria in 2010 [1]. The disease also has severe effects on pregnant women [2] and people living with HIV [3]. The Ghana Ministry of Health describes the disease as the leading cause of morbidity and mortality in the country, accounting for more than one-third of all outpatient cases reported each year, 20 percent to 30 percent of deaths in children younger than five, and 11 percent of maternal deaths [4]. While no malaria vaccine has ever been licensed for use, the global health community is working toward a 2015 time frame for a policy recommendation for a first-generation product that could be deployed for use with existing interventions to help protect young children against the deadly *Plasmodium falciparum* malaria parasite [5].

In order to ensure timely access to such a vaccine, some African countries have begun to build a base of evidence that can help to accelerate national decision-making, so that when a malaria vaccine is recommended for use, countries are also prepared to make decisions on whether to introduce a particular vaccine to complement the existing arsenal of malaria control tools [5], [6]. African policymakers and their international partners have identified the need for a range of data to inform the decision-making process, including data on perceptions of vaccines and malaria [7]. Data on community perceptions would also inform the development of a communications strategy to help ensure that the introduction of a partially efficacious malaria vaccine [8] does not jeopardize continued use of current malaria control measures or reduce confidence in vaccines for other diseases currently in use. Other qualitative studies on community perceptions of malaria and vaccines have been conducted, including in Kenya [9], [10], Mozambique [11], Burkina Faso [12], and in a malaria vaccine trial setting in Ghana, Kintampo [13]. This article provides further data from two non-trial settings in different provinces of Ghana. The study was guided by the following research questions:

1. How is malaria prevention (and treatment) understood and undertaken in the communities?
2. What is the knowledge and acceptance of existing vaccines in the study communities? How do they affect vaccination practices?
3. What are the characteristics of the discourse in the communities about the possibility of a malaria vaccine?
4. What is the decision-making process with regard to child health within families in the study?
5. What audiences and communication channels have been used in previous health information campaigns? Which were the most successful campaigns?

### Ghana malaria and immunization context

Ghana is among the ten countries in the world with the highest malaria morbidity rates and among the 15 with the highest malaria mortality rates [1]. Since the 1950s, the Ministry of Health and the Ghana Health Service have adopted various strategies to control malaria in the population, with special emphasis on children younger than five and pregnant women. These strategies include the distribution of insecticide-treated bed nets door to door (including hanging of the nets), and through schools, antenatal care, child welfare clinics, and public health institutions. Strategies also include the adoption of the Home Management of Malaria program for children younger than five and the use of intermittent preventive treatment of malaria in pregnancy (IPTp) [4]. According to the 2011 Multiple Indicator Cluster Survey, only 51 percent of households owned a mosquito net (whether treated or untreated), and only 39 percent of children younger than five and 33 percent of pregnant women slept under a treated mosquito net the night before the survey [14].

The Expanded Programme on Immunization (EPI) was introduced in Ghana in 1978. In accordance with the World Health Organization and United Nations Children’s Fund guidelines for vaccinating children, the Ghana EPI calendar includes seven types of vaccines for 11 diseases (Table 1). Of these, vaccines for rotavirus and pneumococcal disease and a booster for measles were introduced in 2012. Vaccine coverage in Ghana is among the highest in sub-Saharan Africa. The percentage of children ages 12 months to 23 months who are fully vaccinated (against tuberculosis [with Bacillus Calmette-Guérin], diphtheria-tetanus-pertussis, polio, and measles) increased in the past 20 years in Ghana from 47 percent in 1988 to 84 percent in 2011 [14], [15]. EPI is conducted mainly through programs of the Child Welfare Clinic (CWCs), which are operated at established health centers or hospitals and at outreach clinics. Intensive campaigns are also organized to improve immunization rates and to respond to disease outbreaks. Two immunization campaigns were held in 2012, prior to the start of data collection for this study: the national immunization days for polio in March and May [16] and emergency vaccination in the Upper East Region during the same period, following a cerebrospinal meningitis (CSM) outbreak that caused 23 deaths in the first two months of the year [17]. A CSM mass vaccination campaign for meningococcal meningitis in the three northern regions in October followed the first phase of data collection for this study [18].

**Table 1:** Ghana vaccination calendar 2013.

Month	0	1	2	3	9	18
Visit to CWC	1^st^ visit	2^nd^ visit	3^rd^ visit	4^th^ visit	10^th^ visit	19^th^ visit
**BCG**	♦^1^					
**Polio**	•^1^	•	•	•		
**Pentavalent** 2		▪	▪	▪		
**Pneumococcal**		▪^3^	▪^3^	▪^3^		
**Rotavirus**		•^3^	•^3^			
**Measles**					▪	▪^3^
**Yellow fever**					▪	

BCG: Bacillus Calmette-Guérin for tuberculosis; CWC: Child Welfare Clinic.

Administration path: ♦ Intradermic, • Oral, ▪ Intramuscular.

1 If the baby was delivered in a hospital/health center, it is given at birth.

2 Pentavalent includes diphtheria-tetanus-pertussis (DTP), hepatitis B, and *Haemophilus influenzae* type b. It was introduced in 2002 as a substitute to DTP.

3 Introduced in February 2012 (second dose of measles) and May 2012 (rotavirus and pneumococcal disease).

Ghana is one of a few countries in Africa that has successfully implemented a health insurance system. The National Health Insurance Scheme (NHIS), which covers primary care services and the cost of a range of basic drugs, has intentionally low fees, including a yearly registration fee of two USD for individuals younger than 18 years (if both parents are subscribed), people older than 70 years, and the needy. Pregnant women are exempt from any financial contribution to the NHIS [19]. CWC visits, including routine vaccines, are free for all children, even if they are not registered in the NHIS.

## Methods

### Sites

Two districts were purposively selected for this study. The selection took into account the important ethnic, religious, geographic, climatic, and malaria transmission heterogeneity of Ghana.

In the district selected for the Ashanti Region, located in a southern area of tropical forest, the majority of the population is Asante (Akan), the country’s main ethnic group [20]. They are also Christian and year-round subsistence farmers. In the district selected for the Upper East Region–a savannah area at the border with Burkina Faso–the predominant ethnic group is Frafra (Mole Dagbon), the country’s second main ethnic group [20]. In contrast to the Ashanti district, this area has a more heterogeneous religious profile, including Christianity (mainly Catholicism), Islam, and Animism. As opposed to year-round farming, the single farming season coincides with the rainy season from March/April to September/October. The rest of the year, part of the population migrates to other regions.

In general, malaria in Ghana is hyperendemic, with year-round transmission. However, there is a pronounced seasonal variation in the northern part of the country, where the typical duration of the intense malaria transmission season is about seven months, coinciding with the rainy season [21]. Malaria transmission is therefore more seasonal in the Upper East Region site than in the Ashanti Region site.

Noteworthy public health interventions in the Upper East Region referenced by study participants included the vaccination campaigns for CSM held in 2012 [17], [18]; the filariasis eradication project held between 2001 and 2011 [22]; and the pilot introduction of intermittent preventive treatment of malaria in infants (IPTi) that began with implementation studies in 2007 and continued during data collection for this study [23]. No specific health program of relevance for this research was found in the Ashanti Region.

Within the two districts studied, four urban and rural communities were selected to enhance the diversity of the samples, and the public health facilities that served those communities were also identified. Three hospitals in the Ashanti Region and one hospital and three health centers in the Upper East Region were selected. Semi-structured observations were conducted at the hospitals and CWCs.

### Participant selection and data collection

The qualitative methodology used in this study has its roots in anthropological research. Given the important differences that are known to exist between people’s normative discourses, their actual practices, and their assessment of these practices and knowledge [24], researchers used a diversity of tools to focus both on discourse and behavior:

- **Focus group discussions** permitted a rapid assessment of the normative discourse in the communities regarding malaria and vaccine perceptions. In order to promote practical participatory debate, discussion around vignettes or stories and free-listing and sorting techniques were included in their design (Tables S1, S2).
- **In-depth interviews** allowed researchers to gain deeper insight into the attitudes, knowledge, contradictions, and gaps in information of different participants. These interviews also provided preliminary data on malaria and vaccination practices within the community (Tables S3, S4, S5, S6).
- **Semi-structured observations** added data on practical matters that did not surface in interviews or discussions, perhaps because they were considered too obvious for discussion or seemed to contradict the normative views. Research assistants observed the interactions during the provision of routine childhood immunization services and wrote ethnographic notes on previously defined themes (Table S7).

The triangulation of data from interviews, group discussions, and observations allowed for deeper insight into community members’ perceptions related to vaccinations and their actual practices and experiences at immunization clinics. This approach also permitted the analysis of provider-client communication practices within the context of vaccination services–an important perspective given that community confidence in the quality of vaccine delivery has been shown to influence use of immunization services [25], [26], [27], [28].

A purposive sample (Table S8) was designed for the selection of study participants, using the following criteria: those who make decisions on whether or not to vaccinate young children (parents and other caregivers), and those within communities who influence childhood vaccination decisions (health administrators, health system workers involved in infant and child care, and relevant community members, including teachers, religious leaders, political and traditional authorities, and traditional healers). Mothers of small children were recruited at vaccination clinics and directly in the communities, and some fathers were also selected through previously interviewed mothers (Table 2).

**Table 2:** Study groups and number of collection events.

Tool	Studygroup	AshantiRegion	Upper EastRegion	Total
Groupdiscussions	Mothers	8	6	**14**
	Men/Relevant community members	8	3	**11**
In-depthinterviews	Health administrators	4	4	**8**
	Health professionals	12	6	**18**
	Formal and informal leaders	16	6	**22**
	Mothers	24	15	**39**
	Fathers	12	8	**20**
Semi-structured observations in vaccination clinics		12	9	**21**

Data were collected between May 2012 and mid-January 2013 by four research assistants with experience in qualitative data collection and knowledge of the local languages (Twi and Gruni). Prior to data collection, research assistants were trained, data collection tools piloted, and community stakeholders informed about the research. The translation/transcription of the data collected was completed by the research assistants and double-checked by the site coordinators.

The final sample size across both regions was 286 participants, including 107 in-depth interviewees and 179 in group discussions. This number does not include the semi-structured observations at vaccination clinics, where the number of participants was difficult to determine (Table 2). In the Ashanti Region, all planned collection events were completed. In the Upper East Region, unforeseen delays in the field limited the number of collection events in the scheduled phase (between May and October 2012). In order to achieve conclusive results, preliminary data analysis was conducted in November 2012 to assess the quality and main gaps of the data collected so far, and a short, intensive phase of data collection was held in early January 2013 to ensure the saturation of data for the key points of the research.

Analysis and interpretation were an iterative and flexible part of the anthropological data collection process [29]. Regular meetings occurred throughout the process between those in charge of data collection and those in charge of analysis. These meetings allowed the discussion of perceived relevant themes at the different sites, the development of new questions based on the interpretation of previous data, and the debate of team preconceptions, unexpected findings, contradictions, and doubts. Using Atlas.ti 7, a theme-based, iterative approach was employed to analyze the data. A preliminary list of codes was created based on the topics of the research, and the list was enriched with new categories during analysis. At different stages during the analysis, the research team held discussions in order to clarify and arrive at a consensus on coding, merging categories, and the final comparative analysis and narrative of the findings.

### Ethics statement

Ethical approvals were obtained from the Committee on Human Research, Publication and Ethics of the School of Medical Sciences KNUST, Kumasi, Ghana. ‘Exempt’ approval status was also obtained from the PATH Research Ethics Committee in Seattle, Washington, USA. This meant that the research was deemed exempt from a full research ethics committee review because it fell into a low-risk category, as defined in the US federal regulations. As approved by both ethics committees, participants provided verbal informed consent. Interviewers obtained verbal consent by reading aloud a written script that had been approved by both ethics committees. Verbal rather than written informed consent was preferred in order to avoid the possible negative influence of a written consent on rapport between research assistants and participants. After the agreement of respondents, verbal consent was voice recorded prior to each in-depth interview or focus group discussion.

## Results

### Malaria perceptions

In both regions, study participants considered malaria to be one of the most common health problems among children. Participants differentiated between mild and severe episodes of malaria and described malaria as a very serious disease that could lead to death. Children, followed by pregnant women and the elderly, were considered to be the groups most at risk of contracting malaria.

Study participants used multiple terms to refer to malaria, some of them related to specific symptoms of the disease (Table 3). In the Ashanti Region, the preferred terms were *malaria* and *fever.* While most study participants considered *malaria* and *fever* to be terms for the same condition, they recognized differences between the two. Three contrasting but overlapping meanings were found for *fever*. In some cases, *fever* was associated with a rise in temperature, whereas *malaria* was associated with other perceived symptoms, such as paleness, yellowish eyes, and green vomit. In other cases, *fever* was linked to mild malaria, whereas *malaria* was linked to severe cases. Other participants associated *fever* with yellowness in the eyes and urine. In the Upper East Region, the preferred terms were the local ones that emphasized high temperature (*entolegaban*) and feeling cold from fever (*ooro*).

**Table 3:** Malaria terms.

	Ashanti Region	Upper East Region
**Most frequent**	*Malaria*	*Ooro* (feeling cold from fever)
	*Fever*	*Entolegaban* (high temperature)
	*Whuraye* (paleness)	*Zuwaka* (headache)
	*Atiridee* (yellowish eyes)	*Malaria*
	*Ebunu* (green vomit)	*Fever*
**Least frequent**		*Duusi Ban* (mosquito sickness)

Local terminology and its use revealed that malaria overlapped with different locally recognized conditions in each of the districts. In the Ashanti Region, the main overlap was between malaria, as referred to when talking of *fever* and *atiridee* (yellowish eyes), and yellow fever (commonly called *jaundice*). Both diseases shared descriptions of yellowish eyes and urine. On the other hand, in the Upper East Region, the main overlap was between malaria and pneumonia, as they shared key symptoms used in local terminologies and the cold weather as a cause.

Mosquitoes were considered to be the main cause of malaria, with dirt and stagnant water–associated with mosquito breeding–seen as indirect causes. These two ideas were generally shared with health professionals and conveyed during health talks. In most cases, though, people from the communities did not strictly separate causes of malaria from causes of disease in general. They also did not isolate malaria-specific preventive practices from general hygiene and healthy practices. In this regard, other causes mentioned were the presence of houseflies around food and, in some cases, the eating of very cold food. Participants from the Upper East Region said that wet weather–particularly linked to *ooro*–contributed to malaria. In the Ashanti Region, *malaria*, and especially *fever*, was occasionally associated with the consumption of unripe fruits and oily food.

There was consensus that no one preventive measure stopped malaria transmission completely. At both sites, bed nets were the most cited preventive tool, but certain factors were seen to limit their use. Participants said they were not distributed for free as frequently as they would have liked and in some houses, three or more people shared the same net, making it very hot inside. Participants also cited general hygienic measures, whether indirectly associated with mosquito breeding or not, illustrating the wide range of measures taken for general sickness prevention. While participants occasionally mentioned other malaria prevention strategies such as the use of sprays and coils, price limited the use of sprays, while coils were seen as a fire hazard and linked to some discomfort. In most interviews, it was only when prompted (and not in all cases) that people remembered the preventive use of the drug sulfadoxine-pyrimethamine in pregnant women and the programs in trial areas for IPTi and IPTc (intermittent preventive treatment of malaria in children).

*I(interviewer): Are all (preventive) measures effective? (…)*

*R(respondent)6: It does not matter whether we use these measures we can still get sick because mosquitoes bite us outside even before we sleep so all that we have to do is protect ourselves. (…)*

*R5: When you do all of them, they are effective.*

*R2: When you do them, the malaria can still come but will not be severe. (…)*

*R8: I use both bed net and coil but mosquitoes still bit us.*

*R6: The net is effective but the mosquitoes can still bite us.*

*(Ashanti Region, group discussion with relevant women)*

### Vaccine perceptions

Study participants universally acknowledged vaccination as the main activity conducted within the health system to promote child health. However, the perception of vaccination was closely related to two other concepts: injections and the package of activities at CWCs. The issue was especially evident when people talked about malaria, as treatment in hospitals for severe malaria often includes injections highly valued by community participants. *Pania* in Gruni (Upper East Region) and *panie* in Twi (Ashanti Region) were the words for both vaccine and injection. In order to specify that the conversation was only about vaccines, it was necessary to add the notion of protection in Gruni (*adin gu pania*); whereas, in the Ashanti Region, vaccines were referred to as injections for children (*nkora panie*). The perception was that both vaccines and injections fought children’s diseases–that injections fought diseases that were already “visible,” while vaccines either fought diseases that were “hidden,” or prepared the body to fight in case the disease was coming.

*I: What is your general perception about nkora panie?*

*R: It is good because, when a child has a sickness, the panie is able to take it away…it helps the child to become healthy. (…)*

*I: And what benefits do you get from the nkora paine?*

*R: Nkora panie… the benefit I get is, when the child is sick you will not know, so the vaccines are able to cure the child of the sickness…*

*(Ashanti Region, interview with a mother)*

*I: when the drugs (vaccine) enter into the body, how does it protect the child?*

*R: the drugs poisons the blood so when the virus or the bacteria come, it cannot affect the child.*

*(Ashanti Region, interview with a political leader)*

The understanding of what vaccines do ranged from “fighting diseases” in general to partial knowledge of the diseases they protected against. When discussing the concept of ‘efficacy’, some participants understood that vaccines had helped to eliminate some diseases from the community, whereas other participants saw vaccines as diminishing the severity of the disease.

*R: (…) the kasua (measles) that could have attacked the child and you will lose the child when it is serious, if the child is given this vaccine, this time even if it attacks the child, it cannot get to the state where you lose the child.*

*(Upper East Region, interview with a mother)*

While health professionals directly involved in vaccination programscould name the required vaccines for children in Ghana, this was not the case for other study participants. Some community members could specify a number of them, while others recognized that there were many vaccines. The most common response was somewhere in the middle: while most mothers did not know the specific illnesses the vaccines were for, they could distinguish them through the mode of administration, injection site, or the age of the child at vaccination. As such, differences were seen if the vaccines were given orally or by injection, if they were given in the arm or the leg, or if they were given at one, two, three, or nine months of age.

In naming vaccines against common childhood diseases, the measles vaccine was the most widely known, followed by polio and tetanus. Few study participants mentioned hepatitis B or *Haemophilus* vaccines. Knowledge of vaccines introduced a few months before was slightly higher and was associated with information provided on the radio and at CWCs.

*R3: I took my child to the clinic recently and they injected both left and right thighs that pneumonia and diarrhea. They used to inject only one leg and then, the following month we go back. But they have realized that the diarrhea is disturbing the children a lot that is why they inject the two thighs. That is what they told me before injecting my child.*

*(Upper East Region, group discussion with mothers)*

Participants could also distinguish between vaccines based on whether they were administered at CWCs or through campaigns, as would be the case with polio, CSM, and yellow fever vaccines. When prompted, participants also mentioned vaccines for pregnant women (tetanus) and for adults (campaigns included H1N1 in 2010). In the Upper East Region, participants referred to other non-vaccine prevention activities, such as IPTi and the filariasis eradication program. Vitamin A supplementation was not mentioned.

All study participants acknowledged that vaccines had minor side effects and that these side effects were a negative aspect of an intervention that otherwise had important benefits. Minor side effects were seen to include general discomfort, high body temperature, and swelling. Mothers talked or complained openly about these side effects during semi-structured observations. In addition, health professionals sometimes told mothers what to do in case they occurred, and mothers consulted providers on ways to alleviate them. For the majority of our study participants, these minor side effects were not seen as a reason to stop vaccinating children.

Narratives also suggest that when families associate a vaccine with the occurrence of a serious health problem, they may choose not to have their children vaccinated. In some cases, families seemed to link more serious side effects with questions about the expertise of nurses. For example, in the Ashanti Region, many community members associated vaccines–especially the perceived failure of health professionals to properly administer vaccines–with paralysis. This was evident in the Upper East Region with regard to the CSM vaccine. Participants also linked drugs distributed for filariasis to unspecific, “very serious negative effects” and even deaths. In one example, an Upper East father spoke of “the drug they brought to vaccinate people against elephantiasis”.

*I: Can you mention the vaccine that has these negative effects?*

*R: I have never experienced it in my children but what I have witnessed before is the drug they brought to vaccine people against elephantiasis. For that one, the side effects are nothing to talk about. In fact, I insulted the nurses and prevented my mother from taking it.*

*I: Are some vaccines better than others?*

*R: Yes most vaccines are better than the vaccine for elephantiasis because it disturbed a lot of people. But for the chills and fever in children after vaccinations, it is normal.*

*(Upper East Region, father)*

Rumors were said to have influenced community members’ perceptions of vaccination. However, rumors had been associated mostly with vaccines administered during vaccination campaigns as opposed to vaccines administered as part of established services at CWCs. More specifically, study participants spoke of rumors of deaths related to the H1N1 campaign, a school vaccination campaign for polio in the Ashanti Region, and filariasis drug distribution in the Upper East Region. The radio was one of the main sources for the rumors the participants had heard.

### Child Welfare Clinics

Child Welfare Clinics offer a well-established package of activities for children that includes registration for clinic services, vaccination, the administration of other preventive drugs, weighing, and health talks (Table 4). On occasions mainly related to droughts in the north of the country, CWCs have also been a point for distribution of nutritious food for children. Women are expected to bring their children to the CWC monthly up to the age of five, and the services are free of charge. Perceptions, evaluation, and acceptance of vaccines are strongly linked to experiences at CWCs.

**Table 4:** Child Welfare Clinic structure.

	Waiting area	Registering	Weighing	Vaccination
**Average time** **spent at station.**	Most of the total1–2 hours.	Less than5 minutes.	Less than5 minutes.	Less than5 minutes.
	Health talk lasted10–20 minutes.			
**What happened** **at the station?**	1. Health talk atsome centers.	1. Information wasregistered in RegistrationBooks andChildWelfare Booklets.	All the childrenwere weighed.(In outreachclinics, thisactivity is organized byCBAs.)	Administration ofvaccines, vitamin Asupplementation,IPTi (only in UpperEast Region).
	2. Waiting beforethe activities beganand betweenactivities.	2. Motherswere directedto vaccination area ifnecessary.		
**What health** **information was** **given?**	Health talks aboutbreastfeeding/newhealth programs.	N/A	Comments onwhether the childis growingproperly/advice onbreastfeeding(though not givento every mother).	Partial informationon vaccines andside effects(though notgiven to every mother).
**What discussion** **occurred?**	1. Nurses askedmothers if they hadquestions/doubts.	Discussioncentered onmissed sessions,delayedvisits, and prematurevisits.	Discussion centeredon missed sessions,delayed visits, andpremature visits.	Discussion centeredon missed sessions,delayed visits,and premature visits.
	2. Mothers generallydid not ask manyquestions.			
**What comments did** **mothers have about** **the services at the** **station?**	Mothers saw it as aplace for socialgathering andtrading things forbabies.	Mothers hadno commentson this area.	Weighingappreciated bysome mothers.CWCs areinformally called“weighing.”	Mothers said thatvaccination wasthemain reason forthe clinic visit.
**What comments did** **providers have** **about mothers who** **were at this station?**	Complaints whenpatients did notrespond whencalled.	Complaintsabout missedvisits andpeople usingdifferentCWCs.	No comments.	No comments.
**Average time spent** **discussing** **immunization/child** **vaccine issues.**	Irregular:information wasonly given in themonths around theimplementationof a new vaccine(full health talk).	N/A	N/A	Brief andirregular (lessthan 5 minutes).

CBA: Community-Based Agent; CWC: Child Welfare Clinic; IPTi: intermittent preventive treatment of malaria in infants.

Motivation to attend the clinics was said to include overall appreciation for the value of vaccination and weighing. This appreciation was mixed with a sense of obligation that was reinforced every time women were asked at the CWC to give reasons for missing visits. In addition to the sense of appreciation and obligation, community members stressed that they were motivated by the fact that food was sometimes distributed, especially in the Upper East Region.

CWCs also provided opportunities to socialize and do business. Women dressed in fine clothes and brought money with them, and various businesses were evident. Some people took photos and others sold food and items for babies, including clothes, weighing pants (designed to attach the baby to the scale), baby powder, napkins, and baby bottles. In addition, some people offered pomades, health cards, and protectors for the cards. Nurses at both sites participated in the trading, even though there were references to the fact that they were forbidden from doing business at the CWC.

Most of the costs incurred by mothers during CWC visits were related to socializing. In the Upper East Region and in the urban health centers in the Ashanti Region, some women spent money only on the things they bought around the CWC and in preparing themselves for the visit (e.g., buying clothes and having their hair braided). Some also spent money on transportation, though most women walked to the clinics. Though services were generally supposed to be free, women visiting rural outreach clinics in the Ashanti Region were charged 0.1 USD. The money was provided as an incentive to the Community-Based Agents (CBAs)–volunteers trained by the health system who helped to organize the clinics and who were generally responsible for weighing. In some cases, the money was collected to pay town criers or information centers that helped to mobilize the mothers. All the costs, whether related to socializing or for health services, were seen in the communities as constraints to full CWC attendance.

Time spent at the clinics was another cost considered by mothers. While nurses preferred that women arrive early in the morning, they usually made the first arrivals wait until there were enough women to start the activities. From the structured observations, it was calculated that women who arrived early generally spent between one and two hours at the clinic. When logistical issues claimed the health workers’ attention, some women spent even more time at the CWC.

While women were supposed to go monthly to the CWC for the first five years of the child’s life, most did not go for that long. Significantly, as community members and health professionals noted, parents generally stopped bringing their children to the CWC after the perceived end of the vaccination schedule at nine months. After that, time and cost were given as reasons not to continue with the other CWC activities, which were less valued than vaccines. It was exceptional to find a woman who had continued to bring her child to the CWC for five years.

*I: so at what age are vaccines when given is better off?*

*R: just as you are here talking but usually, from birth, up to nine months. It is usually the last time of giving the children pania. We are supposed to continue sending the children there but I do not do it because of time.*

(Upper East Region, in-depth interview with a mother)

CWC settings offered two opportunities for counseling and providing vaccine information. Nurses delivered a health talk at the beginning of the session or when they considered there were enough women gathered, and an opportunity for more personal counseling came during vaccination. During the semi-structured observations, health talks were given at almost every session in the Ashanti Region, but they occurred in fewer than half of the observations in the Upper East Region. The main topic for health education was child nutrition–exclusive breastfeeding in the first six months and nutritious foods afterward. Other health talks focused on health programs that were being established. For example, pneumococcal and rotavirus vaccines, introduced in May, were topics of some of the health talks observed in June-July. In these talks, nurses explained the conditions vaccines prevented–pneumonia and diarrhea–and their seriousness, and the number of doses and path of administration of the vaccines. They also encouraged women to “take these new vaccines seriously”.

Direct counseling for mothers was brief and irregular, and women tended not to ask many questions. In some cases, the lack of interaction was associated with the heavy workload of nurses who were feeling the pressure to get everything done, and information was generally incomplete. Nurses prioritized instructions on what to do in case side effects occurred. Information on the diseases the vaccines targeted and on the remaining vaccines in the schedule was provided less often. During a majority of the observed vaccinations in the Ashanti Region, the nurses did not provide information on the vaccines they were giving. In the Upper East Region, some women received information on the vaccines, whereas others were only told where on the child’s body the vaccine was to be administered.

During the semi-structured observations, observers noted that nurses often reprimanded mothers for a variety of reasons. Nurses would scold mothers for skipping one or more clinic visits or for giving water to the children when they had been told to breastfeed exclusively. In other situations, nurses would castigate women whom they thought were being impolite. At the same time, none of the mothers complained about nurses being disrespectful. Only some Upper East opinion leaders and Ashanti nurses said the quality of health professionals’ attitudes toward community members deterred women from going to the CWC.

### Malaria vaccine

Even as community members and health professionals agreed that it would be important to have a vaccine against malaria, some participants had the perception that a malaria vaccine already existed. This confusion was associated with limited knowledge of specific vaccines: the perception that vaccines as a whole fought against childhood diseases in general; the perception that there were no clear differences between vaccines and injections, and between vaccines and other preventive strategies such as IPT; and the overlap between malaria and other diseases with fever as a symptom for which there are vaccines available (e.g., yellow fever in the Ashanti Region and pneumonia in the Upper East Region).

*R4: because malaria is rampant, they vaccinate the child the first day you give birth to the child. (…)*

*I: ok. who should be given vaccines?*

*R4: as for vaccination, everybody can be given injection. For example, if I am sick of malaria and goes to the hospital, they give me injection.*

*I: I am talking about injection they give during vaccination period.*

*R4: they normally give those injections to the children.*

*I: what about adults?*

*R4: they don’t give some to adult but they give to the pregnant women. When you go for antenatal they give you malaria vaccination and yellow fever and tetanus.*

*(Ashanti Region, group discussion with relevant women)*

When discussing the possibility of a malaria vaccine with partial efficacy, there was consensus among study participants that people were unlikely to stop using other preventive measures. Data presented so far suggest three reasons for this consensus. Firstly, many participants saw vaccines as only reducing the severity of disease and never providing complete protection. Secondly, community members were already combining different preventive methods–mainly bed nets and hygienic measures–because no single measure was seen as totally efficacious. These factors suggest that community members are open to the use of a combination of methods. Moreover, bed nets and hygienic measures were considered to be good not only for malaria but also to prevent the nuisance of mosquito bites (bed nets) and to prevent many other diseases (hygiene). The prevalent holistic understanding of prevention associated with these two measures suggests communities will continue with them even if there is a malaria vaccine.

*R: the pania is to prevent my child from malaria so it does not mean that I should allow dirt to take over my entire house. So I will continue to clean my home, sleep in the nets with my children and all the rest to prevent other diseases.*

*(Upper East Region, in-depth interview with a mother)*

When discussing the possible mode of administration for a future malaria vaccine, community members indicated that they generally look to health professionals and vaccine developers to decide how vaccines are to be administered. However, when asked about their preferred route of administration, opinion was divided. Some considered injections painful and said that too many vaccines were administered this way. These study participants, most of them in the Ashanti Region, generally preferred the oral method of delivery. Others, including traditional leaders in the Upper East Region, saw injections to be more efficacious, as they go “straight to the blood” and cannot be vomited up, as sometimes happened with “malaria drugs” and the rotavirus vaccine.

Finally, many people asked if a malaria vaccine would be available for adults in addition to children, as is the case with other preventive medicine campaigns. They wondered whether a future malaria vaccine would be introduced in CWCs and only given to children, or if, following the CSM model in the Upper East Region, it would be available for adults as well through campaigns in the communities.

### Communication preferences

Community members highlighted four main avenues for receiving health information: via radio, information vans, health talks, and trusted relevant people in the community. The communication tools least mentioned were posters and television. In urban areas, people preferred radio and information vans, whereas in rural areas, people preferred community durbars (gatherings of chiefs and people of the community), and they trusted their leaders and community health volunteers to provide the relevant information.

Each community described different formal community processes for disseminating health information. In some places, the health system contacted traditional leaders directly, whereas in others, health volunteers (including CBAs) were the ones to deliver the message. Village Health Committees were active in some places but not in others. And while some villages used local information centers to transmit messages, in others, leaders preferred to use the town crier or gong beater. Coordination with the health professionals involved in community sensitization was seen to be necessary in order to determine the best information channel in a given area.

In addition to formal processes, certain informal information networks existed. When deciding on vaccine use, different women stressed that they valued the opinions of other women who had previous experience with the vaccine. Women also said they valued advice on health issues when it came from people involved in the health field, including CBAs, volunteers of previous programs, and traditional birth attendants.

Health administrators and health professionals described the actual strategy of the Ghana Health Service to transfer communication from its headquarters to districts and communities. The picture portrayed seemed to clearly address participants’ preferences: to allow the flexibility needed at the community level; to include communication both with the relevant leaders and, through them, with male heads of households, who had the ultimate responsibility to make decisions; and with mothers, who made the day-to-day decisions, especially those regarding routine trips to the CWC (Table 5).

**Table 5:** Information channels and activities.

Channel	Source		Target audience	Activities
Formalstructures	Ghana Health		Local health	Letters
	Service		professionals	Workshops
			General population	Television
				Radio
				Posters
	Local health		Generalpopulation	Local radiostations
	professionals		(urban)	Vans
			Communities(rural)	Workshops for relevantpeople (e.g.,CBAs and local authorities)
			Mothers (urban and rural)	CWCs: health talksand direct interaction
Communitychannels	Local authorities,CBAs, etc.		Communities(men andwomen)	DurbarsCommunitymeetings
	CBAs, otherrelevant health-relatedpeople		Mothers	Informal interactions

CBA: Community-Based Agent; CWC: Child Welfare Clinic.

## Discussion

The study identified a number of factors that could facilitate the introduction of a licensed malaria vaccine. They include the need to build on the generally positive attitude toward vaccines in the communities, an understanding that malaria prevention requires a comprehensive approach, the availability of well-established programs to deliver new vaccines, the need to increase and improve communication around childhood vaccination, and the identification of flexible, active health communication structures and processes to disseminate information.

### Building on positive attitudes toward vaccines

The findings suggest that communities placed a high value on vaccines in general and were positive about the idea of a malaria vaccine. This positive attitude is the general trend seen in other studies conducted in other districts in Ghana [13] and in other African countries [9]–[12]. Community members acknowledged that malaria was one of the most common and serious diseases, especially among children, and they showed a positive attitude toward new interventions that might help in the control of the disease, as had been previously reported for IPTi in Ghana and other African countries [30] and IPTc [31]. Significantly, study participants considered vaccination the most important activity at the CWCs.

### Understanding that malaria prevention requires a comprehensive approach

Findings suggest that a malaria vaccine would not affect the use of other preventive measures. Firstly, current practices reflect a holistic approach to household health and disease prevention [30], [32], [33] that includes the use of bed nets as well as general hygiene practices, not only for malaria prevention but also to address other health-related concerns–bed nets to address the nuisance of mosquito bites and hygiene practices to prevent multiple diseases. Communities also acknowledged that none of the methods used is totally effective and understood the value of employing different preventive methods at the same time.

Findings also suggest that community members would not see the concept of partial efficacy as new or exclusive to a possible malaria vaccine. While for some, vaccines had been effective in eradicating certain diseases from their communities, for others a disease could be contracted even if a person was vaccinated against it. Community perceptions of vaccines as giving just partial protection were already reported in the 1980s and 1990s in many different countries of Africa and Asia [34]. Findings in this study suggest that this way of understanding the role of vaccines is facilitating more recent community acceptance of new vaccines such as those for pneumococcal disease and rotavirus, and could also facilitate the acceptance of a partially efficacious malaria vaccine in the future.

### Taking advantage of well-established and packaged child welfare programs

The package of services offered at the CWCs appears to provide the best context within which to implement a new vaccine strategy. CWCs are a well-established program that is widely known and respected in communities and regularly attended by mothers and children–at least during the first nine months of a child’s life. On the other hand, rumors were found to influence attendance at vaccination campaigns. The introduction in the last few years of new vaccines and malaria control strategies and the limited number of concerns generated prior to this study could be regarded as an example of how well-established routines and the use of a trusted program considered beneficial by the community can help to ensure the acceptance of new practices [35], [36]. Further, a new intervention would be more likely to be accepted if its administration path, its delivery calendar, and its side effects are similar to those of existing interventions.

### Improving the quality of service and communication around vaccination

Even though CWCs provide a good setting in which to deliver a new vaccine, some limits were observed. Findings in this study show that with most children’s visits ending after the age of nine months, the administration of additional vaccine doses past this time period could pose challenges. Thus, the introduction of the measles booster at 18 months may need to be monitored. The high rates of attendance at CWCs both for antenatal care during pregnancy and during the first nine months of a child’s life show that a monthly routine for pregnant women and newborns could be successful for a limited time before [37] and after birth (Ghana’s EPI coverage is among the highest in Africa [38], [39]). At the same time, a different strategy may need to be found in order to achieve effective attendance after the child’s ninth month.

Even though communities were concerned about side effects and would like full information about the implementation of a new vaccine, data from clinic observations showed that little information was actually provided to caregivers on existing vaccines as part of the routine at the CWCs. Effective communication can close this gap at CWCs, where health professionals have two opportunities to engage with mothers.

The health talk at the beginning of CWC sessions is an opportunity for health workers to provide general information and for mothers, fathers, and other caregivers to share their concerns and ask questions. At this time, vaccine information could be included as one of the regular topics–along with nutrition and breastfeeding–rather than only addressed when a new vaccine has been included on the calendar. Health professionals could also be trained to adjust their communication model. A friendly and dynamic model that community members do not perceive as punitive could be helpful. Strategies could be incorporated to involve mothers in the conversation and to motivate them to express their concerns and ask questions. In regions such as the Upper East, where health talks are not held regularly, it would be important to increase the frequency of these talks.

The personal counseling that takes place while a child is being vaccinated provides another key opportunity for nurse-mother interactions. Observations during this study showed that the workload of the nurses is an important structural factor that has the potential to limit both the quality and length of such interactions. As with the health talks, the focus could be on addressing the specific concerns and questions mothers have rather than on controlling attendance and reprimanding women.

### Identifying a flexible, active health communication structure

A well-established, formal health communications process facilitates the flow of information for a particular strategy from the central offices of the Ghana Health Service to relevant regions and districts, to health professionals involved in direct implementation, and finally to the general public, communities, and caregivers (in the case of an intervention for children). The structure is flexible and allows different approaches in urban and rural areas and is complemented by other community channels that may vary locally. Given the local variability in the community actors involved in distributing health information, the final design of information flows would best be completed in specific local settings, with close attention paid to integrating the views and experiences of health professionals working in the area.

The communication network identified would involve the relevant actors within the context of a vaccination campaign, particularly during the months following the introduction of a new vaccine. However, while CWCs allow for regular direct interaction and communication with mothers, there are no regular activities that target fathers and community leaders, who also play a key role in decision-making related to child health [40], [41]. Regular direct contact between health professionals and community leaders and men that is not limited to specific campaigns could be helpful. Such interactions could help in detecting doubts and rumors and in engaging community discussions related to national decision-making processes, as well as offset the role of radio in the spread of rumors.

### Addressing messaging challenges

In addition to considering all of these factors within the context of vaccine introduction, an effective communications strategy for a future malaria vaccine would take into account certain challenges that relate directly to perceptions of malaria and vaccines. They include:

- **The variety of terms used to refer to malaria** **and their overlap with other biomedical conditions** [42], [43] **.** Similar symptoms are used to identify malaria and yellow fever in the Ashanti Region and malaria and pneumonia in the Upper East Region. Moreover, in the Upper East Region, important differences are evident in the terminology and understanding of malaria even in neighboring areas. Examples can be found in Gruni and neighboring Nankani, where the languages are so closely related that people using one language can perfectly communicate with people using the other. In Gruni, malaria is referred to as *entolegaban* and *ooro*, while Nankani residents mainly use the word *poa*, which refers to a traditional disease in Gruni [43]. Given this variability and overlap, local health workers could be trained to actively include their local knowledge and experience in the final steps of the design of health messages so that they are prepared to communicate with the communities where they work.
- **Partial knowledge of vaccines.** Results have shown that with the exception of a limited number of health workers, study participants could not name the different vaccines given to children. This finding is in line with those presented in previous studies in the Upper East [30] and Ashanti Regions [44], and in Kintampo [13]. These studies also showed that the lack of knowledge is not incompatible with high coverage both of routine vaccines and vaccines given during national immunization days. They also highlighted an expressed interest in vaccinating children against all important childhood diseases, including malaria. Nevertheless, other authors have argued that accurate knowledge and expectations of vaccines and what they do increases the active demand of vaccines. This can be defined as adherence to programs by an informed public who perceives the benefits and need for specific vaccinations, as opposed to passive acceptance or compliance, dependent on authoritative or even coercive recommendations and social pressure [34].
- **Perception that a malaria vaccine already exists.** This understanding is closely related to the two previous points discussed, to the weak distinction between injections and vaccines, and to the use of the malaria preventive measures (e.g., IPTp, IPTi, and IPTc) that are given within antenatal care and with routine childhood vaccines at CWCs. In the design of the health education strategy for a future malaria vaccine, it would be helpful to address the existence of this perception directly, or messages stating its relevance could be easily misunderstood.

### Understanding the overall context for vaccine acceptance

Finally, understanding the context within which communities accept health interventions is also important. In line with previous studies, the results of this study show that final acceptance of new health strategies is more related to the perceived general good and routine acceptance of a package of health activities–such as the CWC and antenatal care–than to the specific knowledge of each intervention [30], [35], [37]. Moreover, there is a general trust in the competence and knowledge of health professionals and health decision-makers [35], [45]. A discussion on the risks of manipulation and misuse of that trust and on the value of full engagement between community members and community-level institutions could be extremely useful.

### Strengths and limitations

Given that qualitative data are always influenced by the interaction between researchers and participants, the study employed both discursive and observational methods in an effort to control and understand these influences. The methods allowed for triangulation and in-depth insight into the relationship between community perceptions of vaccines and community experiences and communication at CWCs. Researchers also used team meetings to discuss linguistic ambiguities and interactions that occurred during field work in order to attune data analysis and interpretation. A different purposive sample design could have allowed find differences in discourse and practices depending on educational level and socioeconomic status The regional differences found in the study highlight two critical points: the importance of careful local planning of communications strategies [46] and the impossibility of generalizing data from two study settings to the whole country.

## Conclusion

This study has identified several factors that may facilitate the introduction of a malaria vaccine. These factors include the highly accepted and well-established routines of the CWCs, the high value placed on vaccination by communities despite their limited knowledge of vaccines, and the shared understanding that malaria prevention requires a comprehensive approach. The finding that communities appeared to be open to using a combination of methods to fight diseases suggests that a partially efficacious malaria vaccine could be part of a tool kit of malaria control strategies. The study has also confirmed the existence of an active and flexible national health communication structure and processes that allow for dissemination and local coordination of messages and activities.

A number of challenges could be addressed through a well-planned communications strategy and well-designed messages. A key challenge to be taken into account is the perception by some community members that a malaria vaccine already exists. This perception is associated with the vague knowledge of vaccines and other preventive strategies. Another challenge is the variety of terms used to refer to malaria and their overlap with other conditions, such as yellow fever and pneumonia. This lack of clarity regarding malaria and its symptoms could have implications for the perceived efficacy of an eventual malaria vaccine. Finally, there is space for reviewing the communications aspects of CWC service delivery to allow for an increase in vaccine information and the use of a dynamic communication model that seeks to engage caregivers of vaccinated children and that community members do not perceive as being punitive. Communicating with mothers about the need to continue CWC visits past the nine-month mark would also be important to ensure administration of any additional vaccination, including the measles booster, which is administered at 18 months.

## Supporting Information

Table S1 Group discussion guide. Malaria.
(DOC)

Table S2 Group discussion guide. Vaccines.
(DOC)

Table S3 In-depth interview guide. Health administrators.
(DOC)

Table S4 In-depth interview guide. Health professionals.
(DOC)

Table S5 In-depth interview guide. Formal and informal leaders.
(DOC)

Table S6 In-depth interview guide. Mothers and fathers.
(DOC)

Table S7 Semi-structured observation guide.
(DOC)

Table S8 Purposive sample per site.
(DOCX)

## References

[1] World Health Organization (WHO) (2012) World Malaria Report 2012. Geneva: WHO.

[2] Desai M, ter Kuile FO, Nosten F, McGready R, Asamoa K, et al (2007) Epidemiology and burden of malaria in pregnancy. Lancet Infect Dis 7: 93–104. 17251080 10.1016/S1473-3099(07)70021-X

[3] Flateau C, LeLoup G, Pialoux G (2011) Consequences of HIV infection on malaria and therapeutic implications: a systematic review. Lancet Infect Dis 11: 541–556. 21700241 10.1016/S1473-3099(11)70031-7

[4] Ghana Ministry of Health (2007) Strategic Plan for Malaria Control in Ghana 2008–2015. Accra: Ghana Ministry of Health.

[5] Malaria Vaccine Funders Group (2013) Malaria Vaccine Technology Roadmap. Available: http://www.who.int/immunization/topics/malaria/vaccine_roadmap/TRM_update_nov13.pdf. Accessed 18 November 2013.

[6] Malaria Vaccine Decision-Making Framework (2012). Available: http://malvacdecision.net. Accessed 18 November 2013.

[7] World Health Organization (WHO) (2005) Vaccine Introduction Guidelines. Geneva: WHO.

[8] Agnandji ST, Lell B, Soulanoudjingar SS, Fernandes JF, Abossolo BP, et al (2011) First results of Phase 3 trial of RTS, S/AS01 malaria vaccine in African children. N Engl J Med 365: 1863–1875. 22007715 10.1056/NEJMoa1102287

[9] Ojakaa DI, Ofware P, Machira YW, Yamo E, Collymore Y, et al (2011) Community perceptions of malaria and vaccines in the South Coast and Busia Regions of Kenya. Malar J 10: 147 10.1186/1475-2875-10-147 21624117 PMC3120733

[10] Ojakaa D, Yamo E, Collymore Y, Ba-Nguz A, Bingham A (2011) Perceptions of malaria and vaccines in Kenya. Hum Vaccin 7: 1096–1099. 21941095 10.4161/hv.7.10.17496

[11] Bingham A, Gaspar F, Lancaster K, Conjera J, Collymore Y, et al (2012) Community perceptions of malaria and vaccines in two districts of Mozambique. Malar J 11: 394 10.1186/1475-2875-11-394 23186030 PMC3519522

[12] Yaya Bocoum F, Kouanda S, Hinson L, Ba-Nguz A, Collymore Y, et al. (2014) Perceptions des communautés sur le paludisme et les vaccins: étude qualitative réalisée dans les districts sanitaires de Kaya et Houndé au Burkina Faso. Glob Health Promot 21: In press. 10.1177/1757975913507729 24496777

[13] Febir LG, Asante KP, Dzorgbo DBS, Senah KA, Letsa T, et al (2013) Community perceptions of a malaria vaccine in the Kintampo districts of Ghana. Malar J 12: 156 10.1186/1475-2875-12-156 23651533 PMC3656774

[14] Ghana Statistical Service (GSS) (2011) Ghana Multiple Indicator Cluster Survey with an Enhanced Malaria Module and Biomarker, 2011, Final Report. Accra: GSS.

[15] Ghana Statistical Service (GSS), Ghana Health Service (GHS), ICF Macro (2009) Ghana Demographic and Health Survey 2008. Accra: GSS, GHS, ICF Macro.

[16] Ghana Health Service (2012) Expanded Programme on Immunization. Upcoming Events. Available: http://www.ghanahealthservice.org/epi_events.php. Accessed 18 November 2013.

[17] International Federation of Red Cross and Red Crescent (IFRC) (2012) Disaster Relief Emergency Fund (DREF) final report. Ghana: Meningitis. Available: http://ifrc.org/docs/Appeals/12/MDRGH006dfr.pdf. Accessed 18 November 2013.

[18] World Health Organization Regional Office for Africa (2012) A mass campaign to vaccinate nearly 3 million people against meningitis “A” starts today in Ghana. Press materials. Available: http://www.afro.who.int/en/ghana/press-materials/item/5013-a-mass-campaign-to-vaccinate-nearly-3-million-people-against-meningitis-%E2%80%9Ca%E2%80%9D-starts-today-in-ghana.html. Accessed 18 November 2013.

[19] Sarapong N, Loag W, Fobil J, Meyer CG, Adu-Sarkodie Y, et al (2010) National health insurance coverage and socio-economic status in a rural district of Ghana. Trop Med Int Health 15: 191–197. 19961565 10.1111/j.1365-3156.2009.02439.x

[20] Service GS (2008) Ghana Population and Housing Census 2000. Accra: Office of the President.

[21] Mara Arma (2000) Mapping Malaria Risk in Africa. Ghana: Duration of the Malaria Transmission Season. Available: www.mara.org.za. Accessed 18 November 2013.

[22] World Health Organization Regional Office for Africa. Ghana Publications (2008) Lymphatic Filariasis elimination (LFE). Available: http://www.afro.who.int/en/ghana/ghana-publications/1728-lymphatic-filariasis-elimination-lfe.html. Accessed 18 November 2013.

[23] Abotsi AK, Inkoom E, Ribaira E, Le Mentec R, Levy P, et al (2012) Cost effectiveness of intermittent preventive treatment of malaria in infants in Ghana. International Journal of Tropical Disease & Health 2: 1–15.

[24] Pool R, Geissler W (2005) Medical Anthropology: Understanding Public Health. London: Open University Press.

[25] Streefland PH, Chowdhury AMR, Ramos-Jimenez P (1999) Quality of vaccination services and social demand for vaccinations in Africa and Asia. Bull World Health Organ 77: 722–730. 10534895 PMC2557734

[26] Jheeta M, Newell J (2008) Childhood vaccination in Africa and Asia: the effects of parents’ knowledge and attitudes. Bull World Health Organ 86: 419–420. 18568264 10.2471/BLT.07.047159 PMC2647458

[27] Andrus JK, Lewis MJ, Goldie SJ, García PJ, Winkler JL, et al (2008) Human papillomavirus vaccine policy and delivery in Latin America and the Caribbean. Vaccine 26: L80–L87 10.1016/j.vaccine.2008.05.040 18945405

[28] Stanton BF (2004) Assessment of relevant cultural considerations is essential for the success of a vaccine. J Health Popul Nutr 22: 286–292. 15609781

[29] Bernard HR (2011) Research Methods in Anthropology. Plymouth: AltaMira Press.

[30] Gysels M, Pell C, Mathanga DP, Adongo P, Odhiambo F, et al (2009) Community response to intermittent preventive treatment of malaria in infants (IPTi) delivered through the expanded programme of immunization in five African settings. Malar J 8: 191 10.1186/1475-2875-8-191 19664250 PMC2734860

[31] Pitt C, Diawara H, Ouédraogo DJ, Diarra S, Kaboré H, et al (2012) Intermittent preventive treatment of malaria in children: a qualitative study of community perceptions and recommendations in Burkina Faso and Mali. PLOS ONE 7: e32900 10.1371/journal.pone.0032900 22412946 PMC3295775

[32] Hausmann Muela S, Muela Ribera J, Mushi AK, Tanner M (2002) Medical syncretism with reference to malaria in a Tanzanian community. Soc Sci Med 55: 403–413. 12144148 10.1016/s0277-9536(01)00179-4

[33] Musoke D, Karani G, Ssempebwa JC, Musoke MB (2013) Integrated approach to malaria prevention at household level in rural communities in Uganda: experiences from a pilot project. Malar J 12: 327 10.1186/1475-2875-12-327 24041445 PMC3848758

[34] Nichter M (1995) Vaccinations in the Third World: a consideration of community demand. Soc Sci Med 41: 617–632. 7502096 10.1016/0277-9536(95)00034-5

[35] Streefland P, Chowdhury AMR, Ramos-Jimenez P (1999) Patterns of vaccination acceptance. Soc Sci Med 49: 1705–1716. 10574240 10.1016/s0277-9536(99)00239-7

[36] Pool R, Munguambe K, Macete E, Aide P, Juma G, et al (2006) Community response to intermittent preventive treatment delivered to infants (IPTi) through the EPI system in Manhiça, Mozambique. Trop Med Int Health 11: 1670–1678. 17054746 10.1111/j.1365-3156.2006.01725.x

[37] Pell C, Meñaca A, Were F, Afrah NA, Chatio S, et al (2013) Factors affecting antenatal care attendance: results from qualitative studies in Ghana, Kenya and Malawi. PLOS ONE 8: e53747 10.1371/journal.pone.0053747 23335973 PMC3546008

[38] Chandramohan D, Webster J, Smith L, Awine T, Owusu-Agyei S, et al (2007) Is the Expanded Programme on Immunisation the most appropriate delivery system for intermittent preventive treatment of malaria in West Africa? Trop Med Int Health 12: 743–750. 17550471 10.1111/j.1365-3156.2007.01844.x

[39] Webster J, Lines J, Bruce J, Armstrong Schellenberg JRM, Hanson K (2005) Which delivery systems reach the poor? A review of equity of coverage of ever-treated nets, never-treated nets and immunization to reduce child mortality in Africa. Lancet Infect Dis 5: 709–717. 16253888 10.1016/S1473-3099(05)70269-3

[40] Tolhurst R, Nyonator FK (2006) Looking within the household: gender roles and responses to malaria in Ghana. Trans R Soc Trop Med Hyg 100: 321–326. 16214194 10.1016/j.trstmh.2005.05.004

[41] Tolhurst R, Amekudzi YP, Nyonator FK, Bertel Squire S, Theobald S (2008) “He will ask why the child gets sick so often”: the gendered dynamics of intra-household bargaining over healthcare for children with fever in the Volta Region of Ghana. Soc Sci Med 66: 1106–1117. 18166257 10.1016/j.socscimed.2007.11.032

[42] Ahorlu C, Koram K, Ahorlu C, de Savigny D, Weiss M (2005) Community concepts of malaria-related illness with and without convulsions in Ghana. Malar J 4: 47 10.1186/1475-2875-4-47 16188023 PMC1262759

[43] Meñaca A, Pell C, Manda-Taylor L, Chatio S, Afrah NA, et al (2013) Local illness concepts and their relevance for the prevention and control of malaria during pregnancy in Ghana, Kenya and Malawi: findings from a comparative qualitative study. Malar J 12: 257 10.1186/1475-2875-12-257 23876079 PMC3724599

[44] Browne EN, Bonney AA, Agyapong FA, Essegbey IT (2002) Factors influencing participation in national immunization days in Kumasi, Ghana. Ann Trop Med Parasitol 96: 93–104. 11989538 10.1179/000349802125000556

[45] Smith L, Jones C, Adjei R, Antwi G, Afrah N, et al (2010) Intermittent screening and treatment versus intermittent preventive treatment of malaria in pregnancy: user acceptability. Malar J 9: 18 10.1186/1475-2875-9-18 20074372 PMC2817700

[46] Cockcroft A, Andersson N, Omer K, Ansari NM, Khan A, et al (2009) One size does not fit all: local determinants of measles vaccination in four districts of Pakistan. BMC Int Health Hum Rights 9: S4. 19828062 10.1186/1472-698X-9-S1-S4 PMC3226236

