# Supplementary material for: Factors Likely to Affect Community Acceptance of a Malaria Vaccine in Two Districts of Ghana: A Qualitative Study
Source: PLoS One. 2014 Oct 15;9(10):e109707. doi: 10.1371/journal.pone.0109707 (PMC4198134; doi:10.1371/journal.pone.0109707)
Supplement: Table S1 — Group discussion guide. Malaria. (DOC) [file pone.0109707.s001.doc]

Table S1. Group discussion guide. Malaria

| **TOPICS** | **QUESTIONS**^1^ |
| --- | --- |
| **Children and infant wellbeing** | ***Vignette***^2^ |
|  |  |
| Main worries and diseases related with children and infant wellbeing | *1. “Kofi / Ama is a 1 year old boy / girl who had been feeling fine and playing happily. One day last week Kofi / Ama woke up crying and his / her mother found that his / her body was very hot”* |
|  |  |
| Decision making regarding children health | What do you think could be Kofi / Ama’s problem? |
|  | What would you do if Kofi / Ama was your own child? |
| Sources of advice in children health | Would you make the decision on what to do on your own, or would you need to discuss it with somebody in the house? With whom? |
|  | Imagine Kofi / Ama’s mother was young and did not know what to do, who could give her good advice? |
|  |  |
|  | *2. “This was on and off for sometime. He / she refused to eat anything. His / her urine was a yellow colour, and after a few hours, he / she vomited”* |
|  |  |
|  | And now, what do you think could be his / her problem? |
|  | What would you do if he / she was your own child? |
|  | Would you make that decision on your own or would you need to discuss it with somebody in the house? Who? |
|  |  |
|  | *3. “A few hours later, he started rolling his eyes. With his eyes opened wide, he was shaking and became stiff”* |
|  | And now, what do you think could be his / her problem? |
|  | What would you do if he / she was your own child? |
|  | Would you make that decision on your own or would you need to discuss it with somebody in the house? Who? |
|  |  |
| **Malaria** |  |
|  |  |
| Wording and images | *From the different diseases that appeared in the previous discussion.* |
|  | Which is the difference between… (examples: fever and malaria, whuraye and malaria, whuraye and fever?) |
|  |  |
| Kinds of malaria (mild and severe) | Are there different kinds of malaria? Which ones? Which are their different symptoms? |
| Symptoms of malaria |  |
|  |  |
| Perceived severity of malaria | Is malaria dangerous? What can happen? Can it kill? |
| Groups more at risk | Are all malaria that dangerous? How can you distinguish those that are dangerous from those that aren’t? |
|  | For who is malaria more dangerous? |
|  |  |
| Care seeking behaviour for malaria | *Referring to the ideas that appeared in the vignette discussion. Use malaria, fever and other terms that appeared* |
| Treatments | *in the conversation* |
| Household management | What are the treatments that could be given at home? Are there home made remedies for some kinds of malaria? |
| Decision making | What are the traditional treatments for malaria? Are they used frequently? When? |
|  | What are the medicines that can be bought from a drug shop for malaria? Are they used frequently? When? |
|  | What are the medicines that are given at the health centre for malaria? Is this option used frequently? When? |
|  | In which other places can you get treatment for malaria? |
|  |  |
| Burden of disease, consequences for the family | When a child is sick with malaria? Who takes care of him in the family? What would that person be doing if the child was not sick? Would the child have been helping someway that he cannot help with malaria? |
|  | How much could it cost to look for treatment for the child? |
|  |  |
| Causes of malaria | *Use the different terms that appeared in the conversation* |
|  | What things can cause malaria? |
|  |  |
| Prevention | What are the measures that can be taken to prevent children to have malaria/fever/…? Are all used at the same time? Why? Which ones are used at the same time? |
|  | Are all the measures effective? Which ones are the most effective? Which ones are the least effective? Why? |
|  | If you are using *(name one of the measure*s)… Can still your children become sick? |
|  |  |
| Previous experience in governmental programs / measures / communication campaigns for malaria | Can you remember different programs for malaria that the government, the hospitals or any other institution has proposed to combat malaria? *(Probe on: community treatment management, bednets distribution, IRS, IPTi, IPTp, new drugs for malaria)* |
|  | Which is your opinion of them? *(Refer of each of the programs cited)* |
|  | Which were the best ones? Which were the worst ones? Why? |
|  | How did you get to know about the program *(radio, health centre talks, other neighbors, community health workers…)* |
|  | Who explained you the program? How was it explained? *(Refer of each of the programs cited)* |
|  | Was there anybody who talked bad about the program? Who? What were they saying? Do you think they were right? Were there people who trusted him? |
|  |  |
| **Malaria Vaccine** | *“As we told you at the beginning (referring to consent form) we have never had a malaria vaccine, but they are now testing one in Ghana and six other African countries. It prevents some episodes from happening but not all, children can still get malaria”* |
|  |  |
| Benefits and limits of the proposed vaccine | Do you think that one vaccine like this could be useful? How? |
| (partial efficacy) | Would you want your children to be vaccinated with this vaccine? Why? |
|  | Would you stop other measures of prevention once your child is vaccinated? Which ones? Why? |
|  | Would you combine it with other methods of prevention? Which ones? Why? |
|  |  |
| Information needed | What would you like to know from this new vaccine before using it? |
|  |  |
| Recommendations for health communication | How should it be presented to the communities? Where would you like to hear about it? |
| on malaria vaccine | Who would you like / trust to talk about the vaccine? |
|  |  |
| Recommendations for its implementation | How do you think it should it be given to children? Where? By whom? At what moment? |
|  |  |

^1^ Some examples of questions for the topic, not an exhaustive list.

**^2^ Adapted from Ahorlu CK, Koram KA, Ahorlu C, de Savigny D, Weiss MG (2005) Community concepts of malaria-related illness with and without convulsions in southern Ghana. *Malaria Journal 4*: 47.**
