# Supplementary material for: Factors Likely to Affect Community Acceptance of a Malaria Vaccine in Two Districts of Ghana: A Qualitative Study
Source: PLoS One. 2014 Oct 15;9(10):e109707. doi: 10.1371/journal.pone.0109707 (PMC4198134; doi:10.1371/journal.pone.0109707)
Supplement: Table S2 — Group discussion guide. Vaccines. (DOC) [file pone.0109707.s002.doc]

**Table S2. Group discussion guide. Vaccines**

| **TOPICS** | **QUESTIONS**^1^ |
| --- | --- |
| **Children and infant wellbeing** | ***Free-listing and sorting*** |
|  |  |
| Main practices to make children grow healthy / prevent diseases | What are the main things mothers / families should do to make their children grow healthy? / to prevent diseases? *(Write them in different papers)* |
|  |  |
|  | *(If vaccination has not appeared probe)* What are the main things that are done in the health centers to make children grow healthy? / to prevent diseases? |
|  |  |
|  | From all these things… *(read the papers you have written)* which is the most important? And the second one? And the third one? *(continue till arrive to the least important)* |
|  |  |
| Decision making regarding children health | Who should make the decision to do these things *(cite things)*? Should the mother discuss it with somebody else before doing them? |
|  |  |
| Sources of advice in children health | Who can advice young mothers when they don’t know what to do to make their children grow healthy? |
|  |  |
| **Vaccines** | *(Probe always asking for examples from their previous experiences)* |
|  |  |
| General perception | What do you think about vaccines? |
|  |  |
| Different kinds of vaccines | Are there different kinds of vaccines? Which ones? |
|  | For which diseases do we have vaccines? |
|  |  |
| Target groups of vaccines | Who should be given vaccines? |
|  | At what age are vaccines given? |
|  | At what age are vaccines better? At what ages are vaccines worse? |
|  |  |
| Benefits of vaccination | Do you vaccinate your children? Why? |
| Negative effects, side effects and | What are the benefits of having your children vaccinated? |
| contraindications of vaccination | Can vaccines have negative effects in the children? Which ones? |
|  | Can vaccines be dangerous? Which ones? Why? |
|  | Are there some vaccines that are better than others? Which ones? Why? |
|  | Are there some moments *(age, contraindications)* when it is better not to vaccine your children? When? Why? |
|  |  |
| Efficacy of vaccines | How much protection does a vaccine give? Do all the vaccines give the same protection? Why? |
|  | Can a vaccinated child still get sick with the disease? Why? |
|  | Does it happen with all the vaccines for different diseases? With which ones is it more common? |
|  | Are there moments when vaccines do not work? Why? |
|  |  |
| How vaccines work | How do vaccines work? |
|  |  |
| Vaccination program perceptions and | How are vaccines given here? Where? When? Who are the responsible for vaccination? |
| experiences: moment, place and people who | Are vaccinations well organized? What can be improved? |
| organizes them |  |
|  |  |
| Obstacles for vaccination | Does everybody in this community vaccine their children? Why? |
|  | Tell me things that prevent people to go to vaccination |
|  |  |
| Decision making processes related to vaccines | Who should make the decision to go and when to go to vaccination? |
|  |  |
| Experience with new vaccines and | In the ten years have there been new vaccines that have been introduced? Which ones? |
| communication campaigns | What do you think of these new vaccines? |
|  | How did you get to know about these vaccines? Who give you the information? |
|  | Did somebody recommend you the new vaccine *(use the names as they appeared in the conversation)*? Who? |
|  | Have you heard negative opinions about these vaccines? Which ones? From whom? |
|  | Have there been problems in the community with the introduction of any of these new vaccines? How? |
|  |  |
| Sources of information related to vaccination | If you have doubts about vaccination, who do you ask about it? |
| and new vaccines | If a young mother has doubts about vaccination, who should she ask about it? |
|  | If you want to know more about a new vaccine? Who would you ask? |
|  |  |
| Diseases they would like to have vaccines for | For what diseases would you like to have new vaccines? Why? |
|  |  |
| **Malaria Vaccine** | *“As we told you at the beginning (referring to consent form) we have never had a malaria vaccine, but they are now testing one in Ghana and six other African countries. It prevents some episodes from happening but not all, children can still get malaria”* |
|  |  |
| Benefits and limits of the proposed vaccine | Do you think that one vaccine like this could be useful? How? |
| (partial efficacy) | Would you want your children to be vaccinated with this vaccine? Why? |
|  | Would you stop other measures of prevention once your child is vaccinated? Which ones? Why? |
|  | Would you combine it with other methods of prevention? Which ones? Why? |
|  |  |
| Information needed | What would you like to know from this new vaccine before using it? |
|  |  |
| Recommendations for health communication | How should it be presented to the communities? Where would you like to hear about it? |
| on malaria vaccine | Who would you like / trust to talk about the vaccine? |
|  |  |
| Recommendations for its implementation | How do you think it should it be given to children? Where? By whom? At what moment? |
|  |  |

^1^ Some examples of questions for the topic, not an exhaustive list.
