# Supplementary material for: Factors Likely to Affect Community Acceptance of a Malaria Vaccine in Two Districts of Ghana: A Qualitative Study
Source: PLoS One. 2014 Oct 15;9(10):e109707. doi: 10.1371/journal.pone.0109707 (PMC4198134; doi:10.1371/journal.pone.0109707)
Supplement: Table S3 — In-depth interview guide. Health administrators. (DOC) [file pone.0109707.s003.doc]

**Table S3. In-depth interview guide. Health administrators**

| **TOPICS** | **QUESTIONS**^1^ |
| --- | --- |
| **Children and infant wellbeing** |  |
|  |  |
| Main worries and diseases related with children and infant wellbeing | Which are the main diseases included in the program for children under five in this district? |
|  |  |
| Main practices to make children grow healthy / prevent diseases | Which are the main prevention programs for children under five in this district? |
|  |  |
| **Malaria** |  |
|  |  |
| Previous experience in governmental programs | Which are the different programs that have been organized for malaria? |
| / measures / communication campaigns for | For its treatment? |
| malaria | For its prevention? |
|  | *(Probe on: community treatment management, bednets distribution, IRS, IPTi, IPTp, new drugs for malaria)* |
|  |  |
|  | Which is your opinion of them? *(Refer of each of the programs cited)* |
|  | Which were the best ones? Which were the worst ones? Why? |
|  | Which are the ones that are working at this moment? |
|  | Which are the ones that have been stopped? |
|  | Is there anyone that will be implemented soon? |
|  |  |
|  | *(Refer of each of the programs cited)* |
|  | How were you informed about the program? |
|  | How was the communication campaign for the program? What materials were used? |
|  | How was the reaction in the communities? Where there negative rumors about the program? In your opinion which was the best communication campaign? Why? |
|  | Which are the new measures that you think should be taken for malaria? |
|  |  |
| **Vaccines** |  |
|  |  |
| General perception | What do you think about vaccines? |
|  |  |
| Different kinds of vaccines | Which are the vaccines that you offer in this district? Are they offered for free? |
| Target groups of vaccines | Who is the target group of each vaccine? |
|  | Are there other vaccines that you think should also be offered? |
|  |  |
| Benefits of vaccination | What are the benefits of having children vaccinated? |
| Negative effects, side effects and | Can vaccines have negative effects in the children? Which ones? |
| contraindications of vaccination | Can vaccines be dangerous? Which ones? Why? |
|  | Are there some vaccines that are better than others? Which ones? Why? |
|  | Are there some moments *(age, contraindications)* when it is better not to vaccine the children? When? Why? |
|  |  |
| Efficacy of vaccines | How much protection do vaccines give to the children? |
|  | Can a vaccinated child still get sick with the disease? Why? |
|  | Does it happen with all the vaccines for different diseases? With which ones is it more common? |
|  | Are there moments when vaccines do not work? Why? |
|  |  |
| Vaccination program perceptions and | How are vaccines given here? Where? When? Who are the responsible for vaccination? Who else is involved? |
| experiences: moment, place and people who | *(Ask them to explain it in detail)* |
| organizes them | Are vaccination programs accepted by the communities? What can be improved? |
|  |  |
| Obstacles for vaccination | Does everybody in this community vaccine their children? Why? |
| Decision making processes related to vaccines | Tell me things that prevent people to go to vaccination |
|  | Who are the people more reluctant with vaccination? |
|  |  |
| Experience with new vaccines and | In the last ten years have there been new vaccines that have been introduced? Which ones? |
| communication campaigns | What do you think of these new vaccines? |
|  | How did you get to know about these vaccines? |
|  | How was the information of the new vaccines given to the communities? *(radio, health talks, sensitizations in the communities, etc)* |
|  | Have there been problems in the community with the introduction of any of these new vaccines? How? |
|  |  |
| Diseases they would like to have vaccines for | For what diseases would you like to have new vaccines? Why? |
|  |  |
| **Malaria Vaccine** | *“As we told you at the beginning (referring to consent form) we have never had a malaria vaccine, but they are now testing one in Ghana and six other African countries. It prevents some episodes from happening but not all, children can still get malaria”* |
|  |  |
| Benefits and limits of the proposed vaccine | Do you think that one vaccine like this could be useful? How? |
| (partial efficacy) | Would you like if it is offered in your district / facility? Why? |
|  | Would you recommend to stop other measures of prevention once your child is vaccinated? Which ones? Why? |
|  | Would you recommend to combine it with other methods of prevention? Which ones? Why? |
|  |  |
| Information needed | What would you like to know from this new vaccine before applying it? |
|  | What should be told about this vaccine to the communities before/when starting to use it? |
|  |  |
| Recommendations for health communication | Which is the best way to give health professionals the information about this vaccine? |
| on malaria vaccine | How should it be presented to the communities? |
|  |  |
| Recommendations for its implementation | How do you think it should it be implemented? |
|  |  |

^1^ Some examples of questions for the topic, not an exhaustive list.
