# Supplementary material for: Factors Likely to Affect Community Acceptance of a Malaria Vaccine in Two Districts of Ghana: A Qualitative Study
Source: PLoS One. 2014 Oct 15;9(10):e109707. doi: 10.1371/journal.pone.0109707 (PMC4198134; doi:10.1371/journal.pone.0109707)
Supplement: Table S4 — In-depth interview guide. Health professionals. (DOC) [file pone.0109707.s004.doc]

Table S4. In-depth interview guide. Health professionals

| **TOPICS** | **QUESTIONS**^1^ |
| --- | --- |
| **Children and infant wellbeing** |  |
|  |  |
| Main worries and diseases related with children and infant wellbeing | What are the main diseases that can happen to children? |
|  |  |
| Main practices to make children grow healthy / | What are the main thing families should do to make their children grow healthy? To prevent diseases? |
| prevent diseases | What are the main things that are done at the health centre to make children grow healthy / prevent diseases? |
|  |  |
| Decision making regarding children health | Who in the family should make the decisions when a child is sick? |
| Sources of advice in children health | Who should a young woman ask for advice when her child is sick and she doesn’t know what to do? |
|  | Who should a young woman ask for advice to make her children grow healthy? |
|  |  |
| **Malaria** |  |
|  |  |
| Wording and images | What are the different words used in the community for malaria? |
|  |  |
| Kinds of malaria (mild and severe) | Which are the groups of people for whom malaria is more dangerous? |
| Symptoms of malaria | Which are the main symptoms of malaria in children? |
| Perceived severity of malaria | Which are the differences between a mild and a severe malaria? |
| Groups more at risk | What should families do when their children have mild malaria? What should they do when they have a severe |
| Care seeking behaviour for malaria | one? What do they do? |
|  | Have you had many cases of children who died from malaria? Can you explain it to me? |
|  |  |
| Treatments | Do you know the home remedies that are used in these communities for malaria? Can you explain them to me? |
|  | Do you know the traditional medicines that are used in these communities for malaria? Can you explain them to me? |
|  | What are the medicines that can be bought from a drug shop for malaria? Are they used frequently? When? |
|  | In which other places can you get treatment for malaria? |
|  |  |
| Decision making | Do you know who in the family makes the decisions in malaria cases? |
| Burden of disease, consequences for the family | Do you know when a child is sick with malaria… Who takes care of him in the family? |
|  | How much could it cost to look for treatment for the child? |
|  |  |
| Prevention | What are the measures that can be taken to prevent children to have malaria? |
|  | Are all used at the same time? Why? Which ones are used at the same time? |
|  | Are all the measures effective? Which ones are the most effective? Which ones are the least effective? Why? |
|  |  |
| Previous experience in governmental programs / measures / communication | Which are the different programs that have been organized for malaria? *(Probe on: community treatment management, bednets distribution, IRS, IPTi, IPTp, new drugs for malaria)* |
| campaigns for malaria | Which is your opinion of them? *(Refer of each of the programs cited)* |
|  | Which were the best ones? Which were the worst ones? Why? |
|  | How did you get to know about the program *(trainings from which institution, radio, colleagues…)*? |
|  | How was the communication campaign for the program? What materials were used? *(Refer of each of the programs cited)* |
|  | How was the reaction in the communities? Where there negative rumors about the program? *(Refer of each of the programs cited)* |
|  | In your opinion which was the best communication campaign? Why? |
|  |  |
| **Vaccines** |  |
|  |  |
| General perception | What do you think about vaccines? |
|  |  |
| Different kinds of vaccines | Which are the vaccines that you offer in this health centre? |
| Target groups of vaccines | Who is the target group of each vaccine? |
|  | Are there other vaccines that you think should also be offered? |
|  |  |
| Benefits of vaccination | What are the benefits of having children vaccinated? |
| Negative effects, side effects and | Can vaccines have negative effects in the children? Which ones? |
| contraindications of vaccination | Can vaccines be dangerous? Which ones? Why? |
|  | Are there some vaccines that are better than others? Which ones? Why? |
|  | Are there some moments *(age, contraindications)* when it is better not to vaccine the children? When? Why? |
|  |  |
| Efficacy of vaccines | How much protection do vaccines give to the children? |
|  | Can a vaccinated child still get sick with the disease? Why? |
|  | Does it happen with all the vaccines for different diseases? With which ones is it more common? |
|  | Are there moments when vaccines do not work? Why? |
|  |  |
| How vaccines work | How do vaccines work? |
|  |  |
| Vaccination program perceptions and | How are vaccines given here? Where? When? Who are the responsible for vaccination? Who else is involved? |
| experiences: moment, place and people who | *(Ask them to explain it in detail)* |
| organizes them | Are vaccination programs accepted by the communities? What can be improved? |
|  |  |
| Obstacles for vaccination | Does everybody in this community vaccine their children? Why? |
| Decision making processes related to vaccines | Tell me things that prevent people to go to vaccination |
|  | Who are the people more reluctant with vaccination? |
|  |  |
| Experience with new vaccines and | In the last ten years have there been new vaccines that have been introduced? Which ones? |
| communication campaigns | What do you think of these new vaccines? |
|  | How did you get to know about these vaccines? Who give you the information? |
|  | How was the information of the new vaccines given to the communities? *(radio, health talks, sensitizations in the communities, etc)* |
|  | Have there been problems in the community with the introduction of any of these new vaccines? How? |
|  |  |
| Diseases they would like to have vaccines for | For what diseases would you like to have new vaccines? Why? |
|  |  |
| **Malaria Vaccine** | *“As we told you at the beginning (referring to consent form) we have never had a malaria vaccine, but they are now testing one in Ghana and six other African countries. It prevents some episodes from happening but not all, children can still get malaria”* |
|  |  |
| Benefits and limits of the proposed vaccine | Do you think that one vaccine like this could be useful? How? |
| (partial efficacy) | Would you like if it is offered from your health centre? Why? |
|  | Would you recommend to stop other measures of prevention once your child is vaccinated? Which ones? Why? |
|  | Would you recommend to combine it with other methods of prevention? Which ones? Why? |
|  |  |
| Information needed | What would you like to know from this new vaccine before applying it? |
|  | What should be told about this vaccine to the communities before/when starting to use it? |
|  |  |
| Recommendations for health communication | Which is the best way to give health professionals the information about this vaccine? |
| on malaria vaccine | How should it be presented to the communities? |
|  |  |
| Recommendations for its implementation | How do you think it should it be given to children? |
|  |  |

^1^ Some examples of questions for the topic, not an exhaustive list.
