# Supplementary material for: Factors Likely to Affect Community Acceptance of a Malaria Vaccine in Two Districts of Ghana: A Qualitative Study
Source: PLoS One. 2014 Oct 15;9(10):e109707. doi: 10.1371/journal.pone.0109707 (PMC4198134; doi:10.1371/journal.pone.0109707)
Supplement: Table S6 — In-depth interview guide. Mothers and fathers. (DOC) [file pone.0109707.s006.doc]

**Table S6. In-depth interview guide. Mothers and fathers**

| **TOPICS** | **QUESTIONS**^1^ |
| --- | --- |
| **Children and infant wellbeing** |  |
|  |  |
| Main worries and diseases related with children | What are the main health problems that can happen to children? |
| and infant wellbeing | What are the main health problems that have happened to your children? |
|  |  |
| Main practices to make children grow healthy / | What are the main things you are doing to make their children grow healthy? To prevent diseases? |
| prevent diseases | What are the main things that are done at the health centre to make children grow healthy / prevent diseases? |
|  |  |
| Decision making regarding children health | Who in the family should make the decisions when a child is sick? |
| Sources of advice in children health | Who *do(es) you / your wife / your wives* ask for advice when one of your children is sick and she doesn’t know what to do? |
|  | Who *do you / does your wife / your wives* ask for advice to make her children grow healthy? |
|  |  |
| **Malaria** | *(This topic would be talked about only in half of the interviews, it has to be talked in detail)* |
|  |  |
| Wording and images | *From the different diseases that appeared in the previous discussion.* |
|  | Which is the difference between… (examples: fever and malaria, whuraye and malaria, whuraye and fever?) |
|  |  |
| Kinds of malaria (mild and severe) | Are there different kinds of malaria? Which ones? Which are their different symptoms? |
| Symptoms of malaria | Have your children suffered them? Which ones? |
|  |  |
| Perceived severity of malaria | Is malaria dangerous? What can happen? Can it kill? |
| Groups more at risk | Are all malarias that dangerous? How can you distinguish those that are dangerous from those that aren’t? |
|  | For who is malaria more dangerous? |
|  |  |
| Care seeking behaviour for malaria | What did you do when your child was suffering *(refer to the episodes discussed before)*? |
| Treatments | What did you do at home? Are there home made remedies for some kinds of malaria? |
| Household management | When did you go to the hospital? What are the medicines that are given at the health centre for malaria? |
| Decision making | What are the traditional treatments for malaria? Did you use them when *(refer to the episodes discussed before)*? |
|  | What are the medicines that can be bought from a drug shop for malaria? Did you use them when *(refer to the episodes discussed before)*? |
|  | In which other places have you ever obtained treatment for malaria? |
|  | Who was involved in making the decisions *(refer to the different steps of the different episodes)*? |
|  |  |
| Burden of disease, consequences for the family | When a child is sick with malaria? Who takes care of him in the family? What would that person be doing if the child was not sick? Would the child have been helping someway that he cannot help with malaria? |
|  | How much could it cost to look for treatment for the child? |
|  |  |
| Causes of malaria | *Use the different terms that appeared in the conversation* |
|  | What things can cause malaria? |
|  |  |
| Prevention | What are the measures that you take at home to prevent children to have malaria/fever/…? Are all used at the same time? Why? Which ones are used at the same time? |
|  | Are all the measures effective? Which ones are the most effective? Which ones are the least effective? Why? |
|  | If you are using *(name one of the measure*s)… Can still your children become sick? |
|  |  |
| Previous experience in governmental programs / measures / communication campaigns for malaria | Can you remember different programs for malaria that the government, the hospitals or any other institution has proposed to combat malaria? *(Probe on: community treatment management, bednets distribution, IRS, IPTi, IPTp, new drugs for malaria)* |
|  | Which is your opinion of them? *(Refer of each of the programs cited)* |
|  | Which were the best ones? Which were the worst ones? Why? |
|  | How did you get to know about the program *(radio, health centre talks, other neighbors, community health workers…)*? |
|  | Who explained you the program? How was it explained? *(Refer of each of the programs cited)*  Was there anybody who talked bad about the program? Who? What were they saying? Do you think they were right? Were there people who trusted him? |
|  |  |
| **Vaccines** | *(This topic would be talked about only in half of the interviews, it has to be talked in detail)* |
|  |  |
| General perception | What do you think about vaccines? |
|  |  |
| Different kinds of vaccines | Are there different kinds of vaccines? Which ones? |
|  | For which diseases do we have vaccines? |
|  |  |
| Target groups of vaccines | Who should be given vaccines? |
|  | At what age are vaccines given? |
|  | At what age are vaccines better? At what ages are vaccines worse? |
|  |  |
| Benefits of vaccination | Do you vaccinate your children? Why? |
| Negative effects, side effects and | What are the benefits of having your children vaccinated? |
| contraindications of vaccination | Can vaccines have negative effects in the children? Which ones? |
|  | Can vaccines be dangerous? Which ones? Why? |
|  | Have you ever had a bad experience with a vaccine? *(If she/he has)* Can you explain it to me? |
|  | Are there some vaccines that are better than others? Which ones? Why? |
|  | Are there some moments *(age, contraindications)* when it is better not to vaccine your children? When? Why? |
|  |  |
| Efficacy of vaccines | How much protection does a vaccine give? Do all the vaccines give the same protection? Why? |
|  | Can a vaccinated child still get sick with the disease? Why? Has it ever happened to your children? Please explain |
|  | Does it happen with all the vaccines for different diseases? With which ones is it more common? |
|  | Are there moments when vaccines do not work? Why? |
|  |  |
| How vaccines work | How do vaccines work? |
|  |  |
| Vaccination program perceptions and | How are vaccines given here? Where? When? Who are the responsible for vaccination? |
| experiences: moment, place and people who | Are vaccinations well organized? What can be improved? |
| organizes them | Please explain to me in detail what happened the last time you took your children for vaccination |
|  |  |
| Obstacles for vaccination | Does everybody in this community vaccine their children? Why? |
|  | Tell me things that prevent people to go to vaccination |
|  |  |
| Decision making processes related to vaccines | Who in your family makes the decision to go and when to go to vaccination? |
|  |  |
| Experience with new vaccines and | In the ten years have there been new vaccines that have been introduced? Which ones? |
| communication campaigns | What do you think of these new vaccines? |
|  | How did you get to know about these vaccines? Who give you the information? |
|  | Did somebody recommend you the new vaccine *(use the names as they appeared in the conversation)*? Who? |
|  | Have you heard negative opinions about these vaccines? Which ones? From whom? |
|  | Did you vaccinate your children with them? How was your experience? |
|  | Have there been problems in the community with the introduction of any of these new vaccines? How? |
|  |  |
| Sources of information related to vaccination | If you have doubts about vaccination, who do you ask about it? |
| and new vaccines | If a young mother has doubts about vaccination, who should she ask about it? |
|  | If you want to know more about a new vaccine? Who would you ask? |
|  |  |
| Diseases they would like to have vaccines | For what diseases would you like to have new vaccines? Why? |
|  |  |
| **Malaria Vaccine** | *“As we told you at the beginning (referring to consent form) we have never had a malaria vaccine, but they are now testing one in Ghana and six other African countries. It prevents some episodes from happening but not all, children can still get malaria* |
|  |  |
| Benefits and limits of the proposed vaccine | Do you think that one vaccine like this could be useful? How? |
| (partial efficacy) | Would you want your children to be vaccinated with this vaccine? Why? |
|  | Would you stop other measures of prevention once your child is vaccinated? Which ones? Why? |
|  | Would you combine it with other methods of prevention? Which ones? Why? |
|  |  |
| Information needed | What would you like to know from this new vaccine before using it? |
|  |  |
| Recommendations for health communication | How should it be presented? Where would you like to hear about it? |
| on malaria vaccine | Who would you like / trust to talk about the vaccine? |
|  |  |
| Recommendations for its implementation | How do you think it should it be given to children? Where? By whom? At what moment? |
|  |  |

^1^ Some examples of questions for the topic, not an exhaustive list.
