# Supplementary material for: Factors Likely to Affect Community Acceptance of a Malaria Vaccine in Two Districts of Ghana: A Qualitative Study
Source: PLoS One. 2014 Oct 15;9(10):e109707. doi: 10.1371/journal.pone.0109707 (PMC4198134; doi:10.1371/journal.pone.0109707)
Supplement: Table S7 — Semi-structured observation guide. (DOC) [file pone.0109707.s007.doc]

**Table S7. Semi-structured observation guide**

| **1. Presentation to the health workers** | Arrive prior to the start of the activities. |
| --- | --- |
|  | Say hello to the health workers, introduce yourself if there is any health worker that was not in previous activities. |
|  | Briefly refresh the information about the study and ask for their permission to follow the activities of the day. |
| **2. Organization of the work** | Observe how the professionals organize their work and interact with the women and children present: |
|  | a. Health professionals involved in the activity |
|  | b. Distribution of tasks |
|  | c. Detailed description of each task |
|  | d. Interaction with the women in each task |
|  | e. Problems of organization commented between the health professionals or observed |
| **3. Health talk** | Detailed description of the information given and the interaction with the mothers present: |
|  | a. Hour of the health talk and professionals involved |
|  | b. Topics talked about and examples presented |
|  | c. Questions asked to the mothers |
|  | d. Questions asked by the mothers |
|  | e. Other kinds of interaction |
| **4. Following 4-8 mothers and child during the process** | Describe the full process followed by a mother with their children from the moment they arrive to the clinic till the moment they leave: |
|  | a. When do they arrive? |
|  | b. Do they need to take a card in the administration? Do they bring the child vaccination card with them? |
|  | c. How do they know the steps they have to follow? |
|  | d. Procedures made to the child |
|  | e. Interaction with the health providers |
|  | f. Interactions between mothers |
|  | g. Do they go to the pharmacy at any moment? |
|  | h. When do they leave? What do they bring with them? |
