# Supplementary material for: Factors Likely to Affect Community Acceptance of a Malaria Vaccine in Two Districts of Ghana: A Qualitative Study
Source: PLoS One. 2014 Oct 15;9(10):e109707. doi: 10.1371/journal.pone.0109707 (PMC4198134; doi:10.1371/journal.pone.0109707)
Supplement: Table S8 — Purposive sample per site. (DOCX) [file pone.0109707.s008.docx]

**Table S8. Purposive sample per site**

| **Group** | Vignette | Mothers (4) | Rural (2) | <25 y.o. (1) |
| --- | --- | --- | --- | --- |
| **Discussion** (16) | for Malaria (8) |  |  | >25 y.o. (1) |
|  |  |  | Urban (2) | <25 y.o. (1) |
|  |  |  |  | >25 y.o. (1) |
|  |  | Fathers (2) | Rural (1) |  |
|  |  |  | Urban (1) |  |
|  |  | Relevant People (2) | Male (1) |  |
|  |  |  | Female (1) |  |
|  | Free Listing and | Mothers (4) | Rural (2) | <25 y.o. (1) |
|  | Sorting for |  |  | >25 y.o. (1) |
|  | Vaccines |  | Urban (2) | <25 y.o. (1) |
|  |  |  |  | >25 y.o. (1) |
|  |  | Fathers (2) | Rural (1) |  |
|  |  |  | Urban (1) |  |
|  |  | Relevant People (2) | Male (1) |  |
|  |  |  | Female (1) |  |
| **In-depth** | Health | District Level (2) | Doctor (1) |  |
| **Interviews** (40) | Administrators |  | Nurse (1) |  |
|  | (4) | Facilities Level (2) | Hospital (1) |  |
|  |  |  | H. Centre (1) |  |
|  | Health | Nurses (6) |  |  |
|  | Professionals | Others involved (3) |  |  |
|  | (12) | Com.H.Workers (3) |  |  |
|  | Formal and | Teachers (4) | Rural (2) | Male (1) |
|  | Informal leaders |  |  | Female (1) |
|  | (16) |  | Urban (2) | Male (1) |
|  |  |  |  | Female (1) |
|  |  | Religious Leaders | Rural (2) | Male (1) |
|  |  | (4) |  | Female (1) |
|  |  |  | Urban (2) | Male (1) |
|  |  |  |  | Female (1) |
|  |  | Traditional / Political | Rural (2) | Male (1) |
|  |  | Leaders (4) |  | Female (1) |
|  |  |  | Urban (2) | Male (1) |
|  |  |  |  | Female (1) |
|  |  | Traditional Healers | Male (1) |  |
|  |  | (2) | Female (1) |  |
|  |  | Others (Identified in | Male (1) |  |
|  |  | Fieldwork) (2) | Female (1) |  |
|  | Mothers (24) | Malaria model | Urban (3) | < 25 y.o. (1) |
|  |  | (12) |  | > 25 y.o. (2) |
|  |  |  | Rural (3) | < 25 y.o. (2) |
|  |  |  |  | > 25 y.o. (1) |
|  |  | Vaccines model | Urban (3) | < 25 y.o. (2) |
|  |  | (12) |  | > 25 y.o. (1) |
|  |  |  | Rural (3) | < 25 y.o. (1) |
|  |  |  |  | > 25 y.o. (2) |
|  | Fathers (12) | Malaria model (6) | Urban (3) |  |
|  |  |  | Rural (3) |  |
|  |  | Vaccine model (6) | Urban (3) |  |
|  |  |  | Rural (3) |  |
